# Infrared Thermography for Monitoring of Freeze Drying Processes—Part 2: Monitoring of Temperature on the Surface and Vertically in Cuvettes during Freeze Drying of a Pharmaceutical Formulation

**DOI:** 10.3390/pharmaceutics14051007

**Published:** 2022-05-07

**Authors:** Håkan Emteborg, Jean Charoud-Got, John Seghers

**Affiliations:** European Commission, Joint Research Centre (JRC), Retieseweg 111, 2440 Geel, Belgium; jean.charoud-got@ec.europa.eu (J.C.-G.); john.seghers@ec.europa.eu (J.S.)

**Keywords:** freeze-drying, lyophilisation, infrared camera, thermography, spatial resolution, pharmaceutical formulation, germanium, soda-lime glass

## Abstract

The coupling of an infrared (IR) camera to a freeze dryer for monitoring of the temperature of a pharmaceutical formulation (sucrose/mannitol solution, 4:1%, *m*/*m*) during freeze-drying has been exploited further. The new development allows monitoring of temperatures simultaneously at the surface as well as vertically, (e.g., in depth) along the side using custom-made cuvettes. The IR camera was placed on the chamber roof of a process-scale freeze dryer. Monitoring of cuvettes containing the formulation took place from above where one side of each cuvette was equipped with a germanium window. The Ge-window was placed next to an IR mirror having a 45° angle. The long-wave infrared radiation (LWIR) coming from the inside of the cuvette was reflected upwards toward the IR camera. Accurate recording of the temperature along the cuvettes’ depth profile was therefore possible. Direct imaging from −40 °C to 30 °C took place every 60 s on the surface and on the side with a 2 × 2 mm resolution per IR pixel for 45 h resulting in 2700 thermograms. Results are presented for freeze-drying of a pharmaceutical formulation as a function of time and spatially for the entire side (depth) of the cuvette. As the sublimation process was progressing, the spatial resolution (84 IR pixels for the side-view and 64 pixels for the surface-view) was more than sufficient to reveal lower temperatures deeper down in the material. The results show that the pharmaceutical formulation (a true solution at the onset) dries irregularly and that the sublimation front does not progress evenly through the material. During secondary drying, potential evaporative cooling of upper layers could be detected thanks to the high thermal and spatial resolution.

## 1. Introduction

Freeze-drying is an excellent way of drying thermally sensitive materials to increase their long-term stability and preserve thermally labile compounds if stored properly afterward. The technique is for that reason widely used in the pharmaceutical industry and for the stabilisation of certified reference materials [1,2,3,4,5,6]. In the pharmaceutical industry, different formulations are used to facilitate freeze-drying of active compounds, e.g., to act as bulking agents to provide a network for the active substances contributing to rapid and complete reconstitution before use. Other agents can be added acting as cryo-protectants, buffers, and stabilisers for the active compounds [7,8,9,10,11]. In this study, a 4:1% (*m*/*m*) solution of mannitol:sucrose in water was freeze-dried as a representative matrix for a pharmaceutical bulking agent and cryo-protectant formulation [7,8,9,10,11].

A freeze-drying cycle consists of three main steps 1, 3, and 4 as given below: Whereas annealing (step 2) is optional.

(1) Freezing of the water present in the matrix at ambient pressure without causing severe phase separation and too small ice-crystals.
(2) Annealing of ice to achieve crystals of larger and uniform size [11].
(3) Sublimation (primary drying), whereby the water is evaporating from solid ice in the material and is captured on the ice-condenser under a soft vacuum.
(4) Secondary drying where most of the remaining water is removed under a hard vacuum and a shelf temperature of around 20 °C.

In an earlier paper, we reported on the first use of combining an infrared camera (IR) with a freeze dryer for monitoring the surface temperature when freeze-drying human serum in vials and cheese slurry on trays [12]. In that work, the major benefits of the IR camera were outlined. The coupling resulted in superior spatial and thermal resolution of the monitored materials in contrast to the use of traditional Pt-100 probes placed directly in the materials. A limitation of the previous setup was that only the surface of the materials could be monitored [12]. In this work, we present a solution so thermal data along depth profiles inside the material could also be recorded using the IR camera.

Several other studies have since then been published describing work using an infrared camera to monitor freeze-drying processes [13,14,15,16,17,18,19]. Lietta et al. described the use of an infrared camera mounted inside a freeze dryer compared with results from thermocouples when drying a sucrose formulation in glass vials. In that work, lateral observation of the glass vials was possible [15]. Contactless techniques are preferred in PAT (Process Analytical Technologies) but Song et al. published a paper to elucidate the spatial temperature distribution inside a material based on physical probes [13,20]. In their work, twenty-four measurement points were systematically placed every 2 × 4 mm inside skimmed milk subjected to freeze-drying. In the current paper, an IR camera coupled to a freeze dryer was exploited further by developing custom-made vials, i.e., 10-mL glass cuvettes with one side made of germanium (Ge). The choice of germanium for one window was based on the fact that this material is almost fully transparent in LWIR 7–14 µm. The remaining walls of the cuvette to be studied were made of modified soda-lime glass, which is similar to normal glass. The size of these recipients was chosen so that they can be representative of drying behaviour in glass vials filled with about 8 mL liquid. By using cuvettes with a Ge-window it was possible to record the temperature vertically along a depth profile inside the cuvette albeit at the material-surface adjacent to the Ge-window. By placing the side made of germanium next to a 45° IR mirror, it was possible to record the temperatures prevailing at the surface as well as deeper down in the material simultaneously. This set-up allowed monitoring of the gradual change of temperature in the cuvette from the top to bottom as a function of time through a full freeze-drying cycle based on 2700 thermograms. The depth profile reached about 24 mm deep inside the cuvette corresponding to the fill height of the pharmaceutical formulation. Two main categories of results are presented: (i) temperatures along one depth profile as a function of time during the complete freeze-drying cycle are shown for freezing, annealing, sublimation, and secondary drying; (ii) contour plots of temperatures are shown for the side (depth) of the cuvette covering 14 × 24 mm of the side at specific time intervals. The spatial contour plots are based on 84 contact-free data points (IR pixels).

To our knowledge, it is the first time germanium vials (monitored by infrared thermography) have been used inside a freeze dryer. Thanks to this, it was possible to show minute differences in spatial and temporal temperature distribution inside these vials during a complete freeze-drying cycle also along depth profiles. When such detailed information is available, further optimisation of the freezing and annealing steps becomes straightforward. This should lead to increased efficiency of the subsequent sublimation step, which ultimately provides more uniformly dried materials of better quality.

## 2. Materials and Methods

An infrared camera (VarioCAM, Jenoptik, InfraTec GmbH, Dresden, Germany) was mounted vertically, with the lens facing downwards (looking through a germanium window) and placed in the vacuum chamber wall of a freeze dryer (Epsilon 2-100D, Martin Christ, Osterode, Germany) [12]. The top shelf that was monitored by the IR camera was coated with masking tape using 50 mm wide strips that had been neatly placed next to each other. The shelf temperature could therefore be directly monitored using the IR camera since the tape layer is drastically increasing the emissivity as previously described [12]. The masking tape was of 140 µm thickness consisting of a slightly creped paper with a rubber adhesive from Tesa type 4316 (Brussels, Belgium). The IR camera recorded images of the material in the Ge-cuvettes as well as the shelf of the freeze dryer with a high spatial and thermal resolution (2 × 2 mm, spatial) and (50 mK, thermal) by measuring long-wavelength infrared radiation (LWIR) from 7 to 14 µm. The outside dimensions of the cuvettes were 38 mm high and 22 mm wide and made of soda-lime B270 optical glass of 2 mm thickness (Hellma, Müllheim, Germany). The cuvettes were supplied with one side missing, onto which an IR transparent germanium window was glued. The germanium windows of 2.2 mm thickness measuring 38 × 22 mm were obtained from Umicore Electro-Optic Materials (Dundee, UK) having an average IR transmission >97% from 7 to 14 µm. The germanium windows were coated with a HEAR/HEAR coating (high-efficiency broad-band anti-reflective coating) of undisclosed composition that resulted in a maximum reflectance of 0.7% from 7 to 14 µm. The Ge-windows were glued onto the glass cuvettes using Fix ALL Crystal based on a modified silane polymer (Soudal, Turnhout, Belgium). This glue is compatible with temperatures from −40 °C to 90 °C. The prism (IR mirror) with a 45° angle, (Umicore Electro-Optic Materials) was placed next to the cuvettes to monitor the side of the cuvettes with the IR camera looking from above. The prism was 200 mm long and 50 mm high and made from N-BK7 borosilicate glass. To achieve >99% reflectance from 7 to 14 µm it was coated with protected gold on the chamfered side. The prism was long enough to accommodate at least five cuvettes standing next to each other along the IR reflecting surface. Furthermore, D-mannitol (>98% pure) and sucrose (>99.5% pure) were obtained from Sigma-Aldrich (Steinheim, Germany) and dissolved in Type-1 water (18.2 MΩ cm, 0.053 μS cm^−1^, Merck Millipore, Billerica, MA, USA) to give a 4:1% mannitol:sucrose solution (*m*/*m*). 

Additional technical details about the coupling of the infrared camera to the freeze-dryer, data acquisition, and performance details can be found in previous work [12]. After drying, the water content of the mannitol/sucrose formulation was measured using volumetric Karl Fischer titration using about 160 mg of dried material per replicate, (Metrohm, Herisau, Switzerland). A CombiTitrant 5 (Aquastar^®^ from Supelco) made by Merck KGaA, Darmstadt, DE was used as titrant where 1 mL corresponds to about 5 mg of water. Methanol Emsure, ACS ISO Reag. Ph Eur, Merck Darmstadt, DE was used to disperse the dried material. To calibrate the titre, a sodium tartrate di-hydrate CRM was used from Honeywell Fluka, Seelze, DE. This CRM was produced under ISO17034 accreditation and controlled against NIST SRM 2890 (Gaithersburg, MD, USA). Refined “Everyday” olive oil was obtained from a grocery store, Colruyt, Mol, BE.

The freeze-drying cycle for the formulation was both controlled and monitored using machine readings of shelf temperatures, product temperatures, and pressure. Product temperature and resistivity readouts (Lyo RX) were obtained from Pt100 probes or Lycontrol probes immersed in the product and connected by wire to the freeze-dryer. In addition, 2700 thermograms were recorded for 45 h. The thermograms were processed afterward and corrected for emissivity in the dedicated IRBIS 3 software from Jenoptik. Temperature data for profiles and other measurement definitions was exported as ASCI files (readable with a text editor), imported into Excel, and then further manipulated in Sigmaplot (v. 14.5) to generate spatial contour plots.

## 3. Results and Discussion

**Figure 1:**
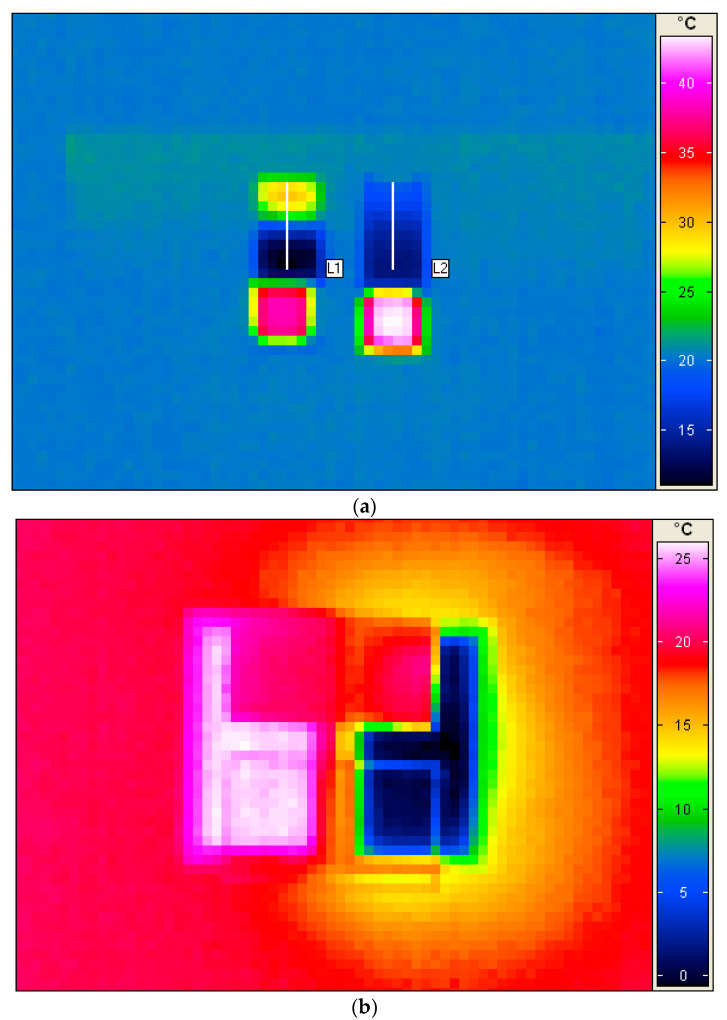

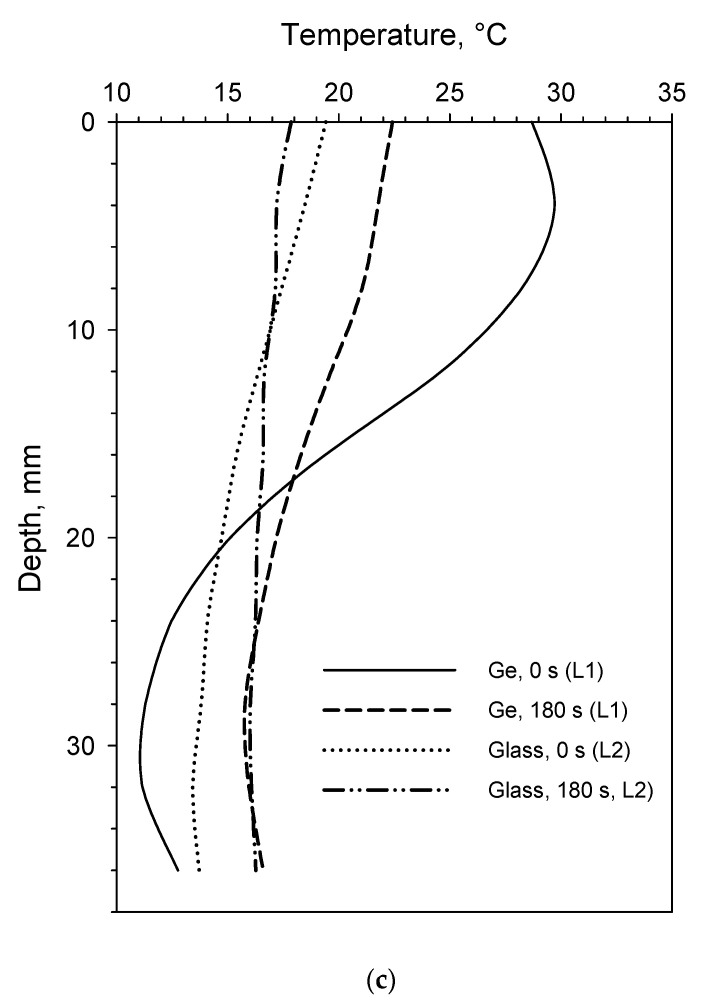
(**a**) Thermograms were recorded from above outside freeze-drier of the two cuvettes (IR mirror vaguely discernible in light green). For the cuvette on the left, the Ge-window was placed next to IR mirror, and on the right; the optical glass window was placed next to IR mirror. Both cuvettes were filled with 7.5 mL ice-water (3 °C) and 2.5 mL warm olive oil (50 °C). The thermogram was taken at 0 s for a 180-s measurement sequence. (Note that this set-up is rotated 180° in comparison with Figure 4a–c). (**b**) Thermogram of B270 soda-lime glass window (top) and germanium window (bottom) placed on top of two recipients filled with 25 °C water (left) and 1 °C ice water (right). A 2-cm gap of air was present between the windows and the water surfaces. (**c**) Temperature at 0 and 180 s, respectively, along depth profiles L1 and L2 in (**a**). The B270 soda-lime glass window is opaque to LWIR transmission in contrast to the Ge-window.

First, the ability of the IR camera to record the temperature accurately through the Ge-windows was verified. Two cuvettes were placed next to the prism at ambient temperature outside the freeze-dryer. One cuvette was placed so that the Ge-window was facing the prism and for the other cuvette, the glass window was facing the prism. The IR camera was placed above the setup looking downwards at cuvettes and prism using a tripod. The cuvettes were filled with 7.5 mL of cold water (3 °C) and immediately afterward, 2.5 mL of warm olive oil was added (50 °C) on top. As soon as the olive oil was added, a three-minute measuring sequence was started by recording one thermogram every ten seconds. From the start, the temperature of the cold water immediately started to increase and the temperature of the oil was rapidly decreasing as a function of time. As can be seen in Figure 1a, the thermogram recorded through the Ge-window shows a large difference between the warm top layer of oil and the colder water at the bottom. The temperatures measured on the glass window (soda-lime B270 optical glass) show a much smaller temperature gradient and it was not possible to record the temperature prevailing inside the cuvette accurately. The glass window is opaque for IR radiation since this type of glass has little transmission in the LWIR range. If glass cuvettes would face the IR mirror in subsequent freeze-drying experiments, it would result in a measurement of the surface temperature of the glass. The temperature of the glass surface is caused by the heating or cooling of the glass from the material inside the cuvette. Such an approach results in a loss of spatial and thermal resolution concerning the actual temperature prevailing *inside* the cuvette. In contrast to this, the flat Ge-windows facilitate accurate temperature measurements at the surface of the material *inside* the custom-made cuvettes. In an additional initial test, the soda-lime glass window and the germanium window were placed over two recipients with cold and warm water, respectively. In this case, there was a gap of air between the water and the windows. The thermogram is shown in Figure 1b. As can be seen, without contact with the water in the recipients, temperature measurements on the soda-lime glass window show the ambient temperature of the glass only. Hence, it is shown that this type of glass has no transmission in LWIR. On the other hand, no significant difference between the temperature read-outs through the germanium window and the water surfaces could be established at *p* 0.05 by doing a *t*-test of the average temperatures. As can be seen in Table 1, there is a large difference in thermal conductivity and IR transmission efficiency between soda-lime glass and germanium in the LWIR range. In fact, only special chalcogen glasses and special ceramics exhibit transmission in LWIR [21,22]. 

**Figure 2:**
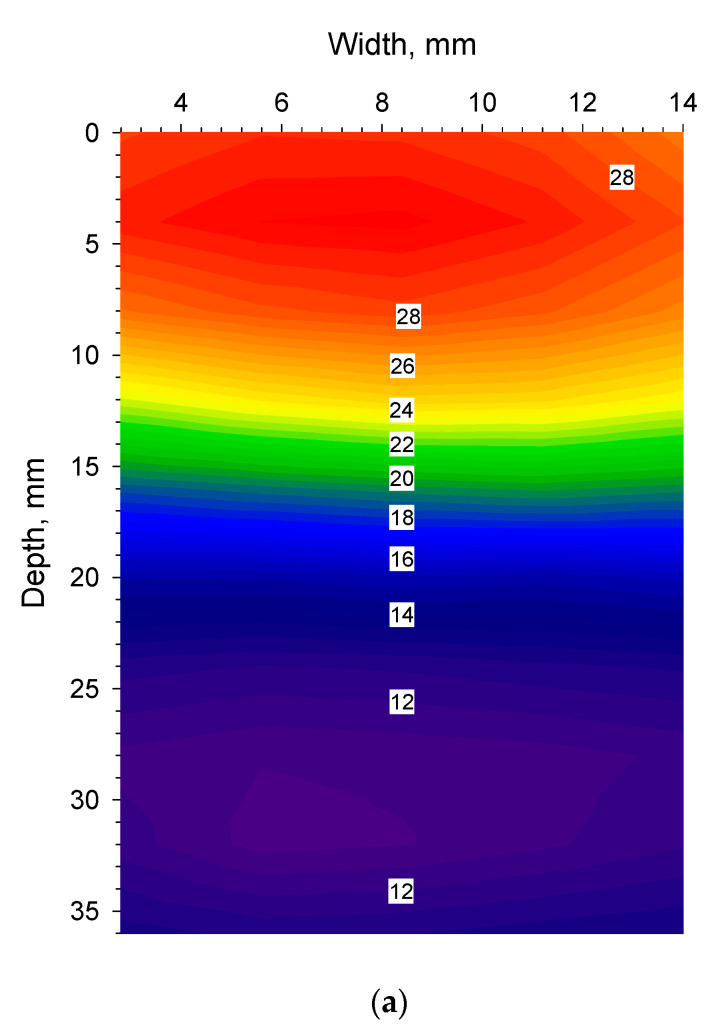

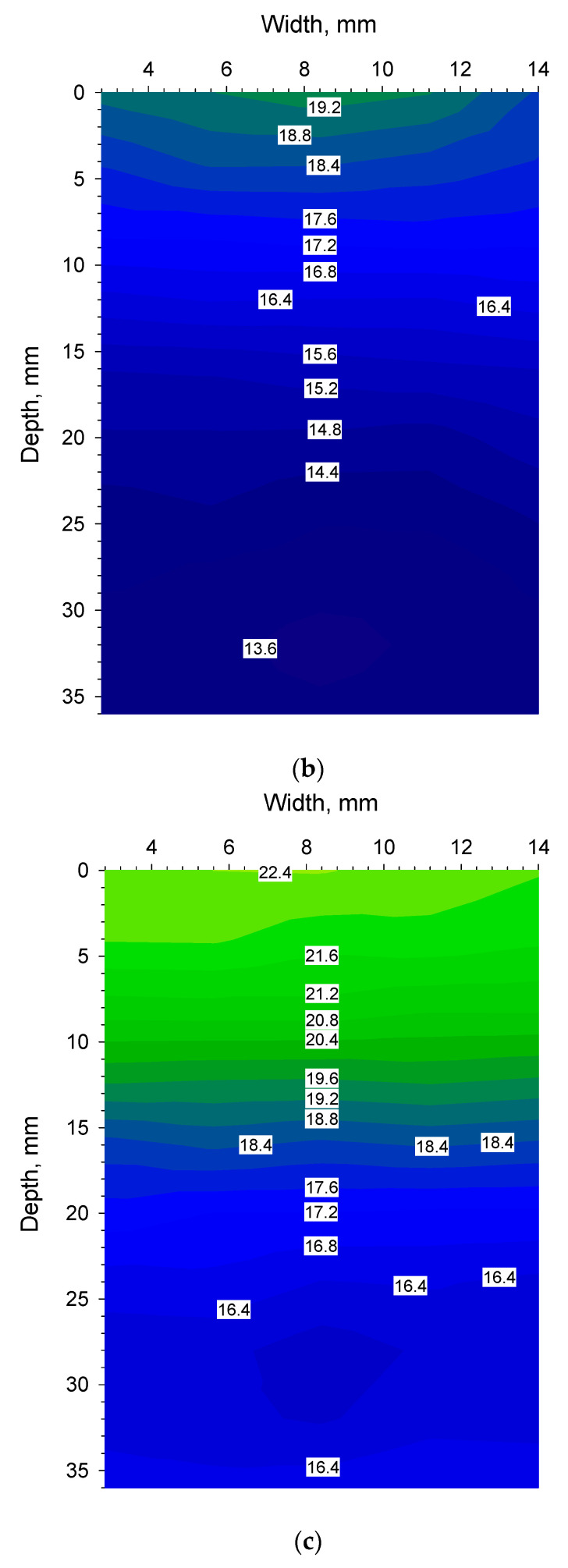

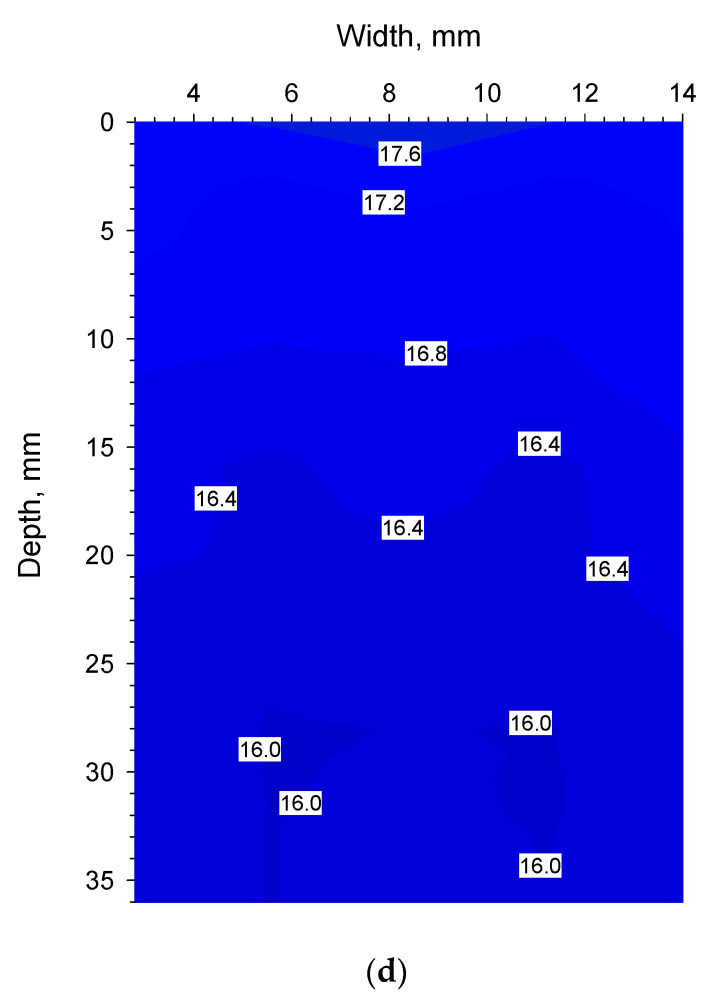
(**a**). Olive oil–water experiment, germanium window monitored after 0 s. (**b**). Olive oil–water experiment, soda-lime glass window monitored after 0 s. (**c**). Olive oil–water experiment, germanium window monitored after 180 s. (**d**). Olive oil–water experiment, soda-lime glass window monitored after 180 s.

**Figure 3:**
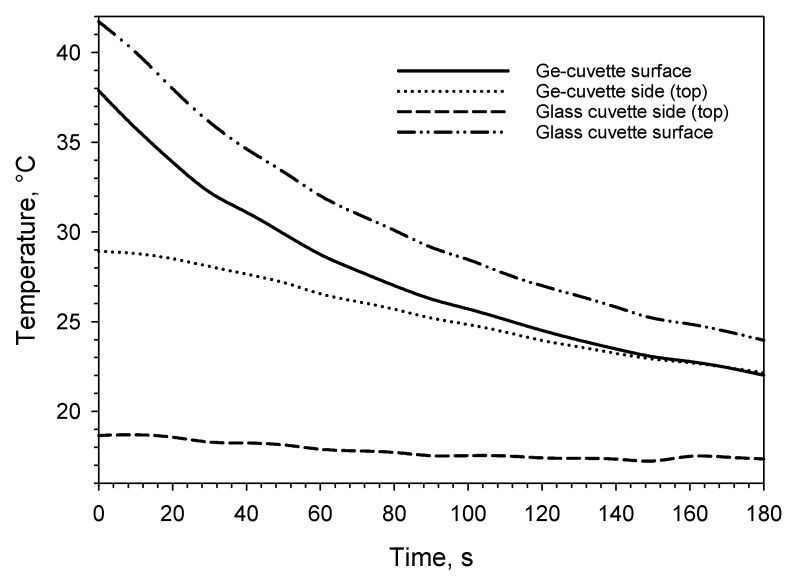
Time profiles of average temperature from ten IR pixels at the surface and ten IR pixels at the side (top) of the two cuvettes as shown in Figure 1a employing measurement areas as shown in Figure 4b. The olive oil is clearly warmer at the *surface* at the beginning of the experiment. After 120 s, the difference is only 0.5 °C between the surface and topside (about 2 mm down in the liquid) through the Ge-window but for the glass window, the difference is still >12 °C between the surface and topside.

One may think that the difference in thermal conductivity is too large between the two materials so that the observations from the drying process recorded with Ge-windows cannot be extrapolated to vials made from glass, as this material is a thermal insulator. In this freeze-drying process, the energy input to push water out of the materials comes from step-wise increments of the shelf temperature. As the Ge-cuvettes have their bottom made of glass as well as three other sides, they are similar to any glass vial in that respect. The energy transfer from the shelf to the material through the bottom of the vial is also similar to a regular glass cuvette/vial. In addition, as shown in Figure 1c, the actual temperatures plotted along the depth profiles of the Ge-cuvette and glass cuvette after 0 and 180 s, show the limitations of monitoring a glass surface with an infrared camera. Nevertheless, it must be also noted as shown in Figure 1c, that the temperature reading of the glass and germanium window is almost identical after 180 s from 24 mm towards the bottom in the cuvette. Van Bockstal et al. reported on the use of an IR camera to monitor rotating glass vials [14]. In another publication, freezing behaviour inside glass vials was also studied using an infrared camera [19]. Correct temperature readings were obtained as also shown here, but without high thermal and spatial resolution to reveal more subtle differences [14,19]. In Figure 2a–d, spatial contour plots of the germanium and glass windows are shown for the oil–water experiment after 0 and 180 s, respectively. The colour coded temperature scale in those figures is the same to facilitate direct comparison. Figure 3 shows the temperatures at the surface and top of the side of the cuvette (1–2 mm below the surface). Admittedly, the temperature changes in the oil–water experiment are generally faster than in a freeze-drying process. At the same time, frozen or drying materials do not necessarily have equally good contact with the cuvette material surface as liquids have (as in this oil–water experiment). This could also be detrimental to the correct readout of the temperature of the glass surface as good material contact is necessary for an accurate temperature reading of the glass surface.

**Figure 4:**
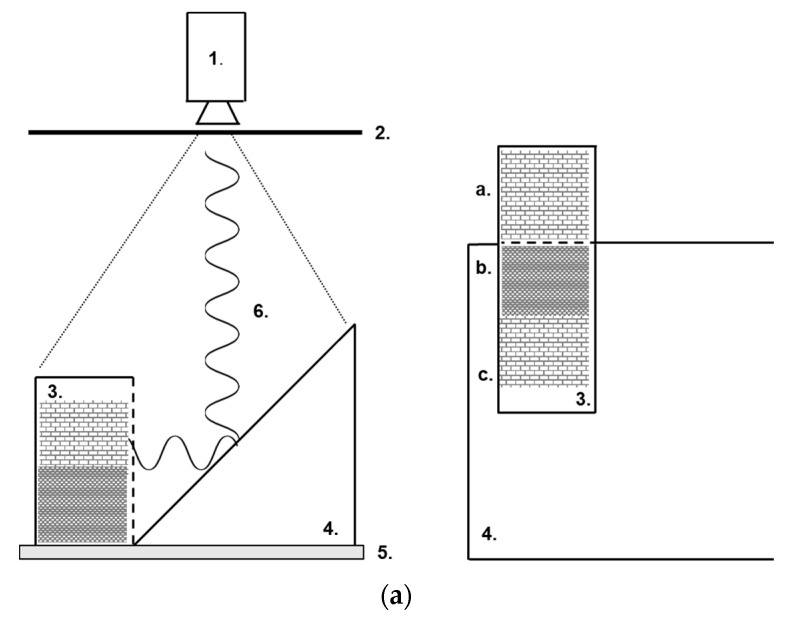

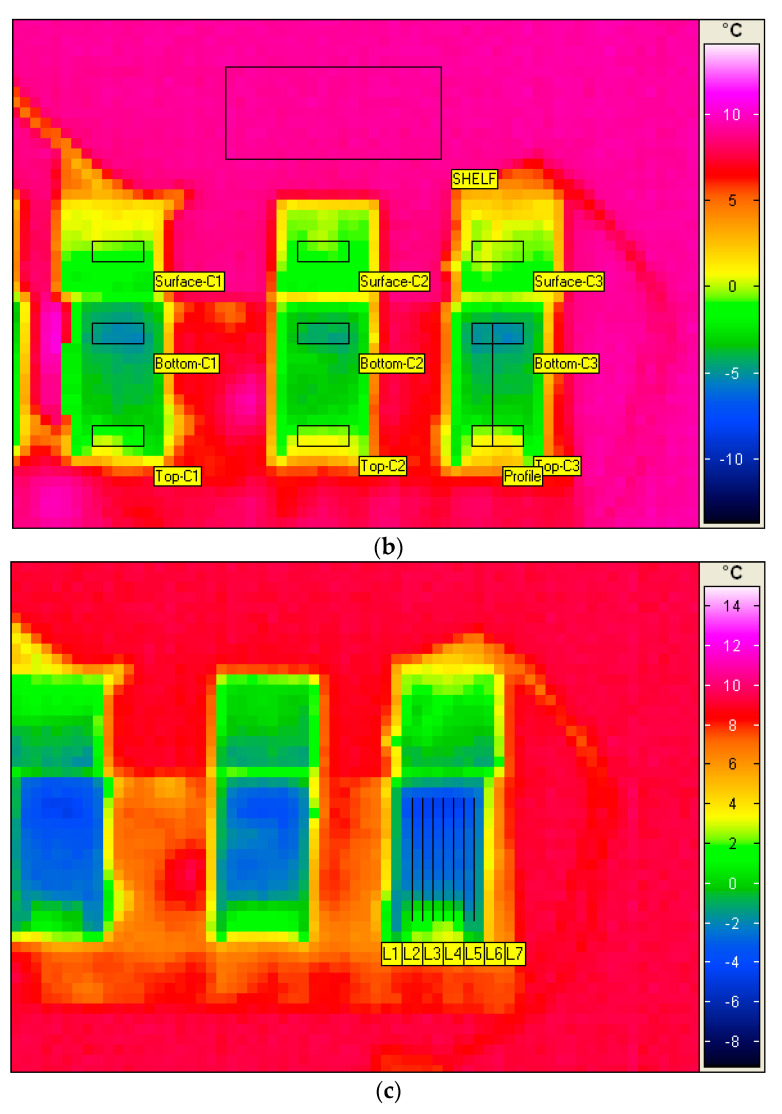
(**a**). Schematic overview to the left, IR camera (1), main Ge-window in chamber-wall of freeze-dryer (2), (pieces 1 and 2 not drawn to scale), side of Ge-cuvette with dashed line indicating Ge-window (3), IR mirror (4) and freeze-drying shelf (5). LWIR radiation recorded by IR camera (6). To the right, reflection in IR mirror as seen by IR camera (4). Direct view of surface of the material in Ge-cuvette where the regular brick pattern is indicating dry material a. Reflection of side of Ge-cuvette (3) where the bottom of the cuvette side is shown as a compressed brick pattern indicating still frozen material b. The top part of the reflection is showing regular brick pattern indicating dry material c. (**b**). Thermogram of three Ge-cuvettes with nine monitoring zones and one depth profile (right). The nine monitoring zones cover 10 pixels each where each pixel is 2 × 2 mm on the measured object. The depth profile covers 12 pixels or 24 mm. Temperatures of all time profiles shown in Figure 6 and Figure 7a–d were monitored using this profile. Monitoring of shelf temperature was performed inside the large rectangle of 198 pixels at the top (SHELF). (**c**). Seven depth profiles used to create spatial contour plots of Ge-window shown in Figure 8a–i. The lines are covering 24 × 14 mm obtaining temperature data from 84 IR pixels. The rims of the cuvettes are clearly visible and were not monitored.

**Figure 5:**
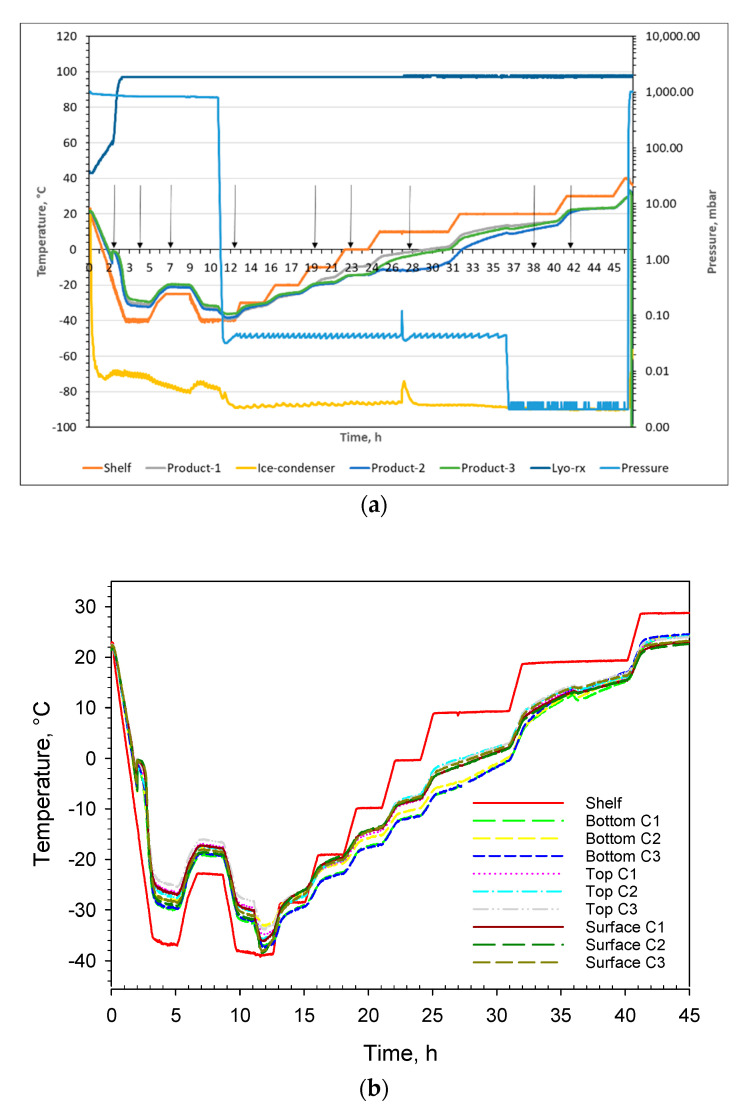
(**a**). Full freeze-drying cycle of the pharmaceutical formulation based on machine readings of product temperature from three Pt-100 sensors, shelf temperature, pressure, ice-condenser temperature, and resistivity (lyo-RX). Features such as super-cooling, annealing, sublimation, and secondary drying are clearly visible. On top, the lyo-RX value shows 95%, which is indicative of frozen material (no melt) during complete cycle after freezing. Vertical arrows indicate time points of spatial images shown in Figure 8a–i. The read-out values are essentially identical to the set values for pressure and shelf temperature as a function of time. The freeze-drying program can therefore be deduced from (**a**). (**b**). Time profile of the different measurement zones defined in Figure 4b with temperature data taken from 2700 thermograms. Temperature profiles are in close alignment with the temperature read-outs from the freeze dryer shown in (**a**).

**Figure 6:**
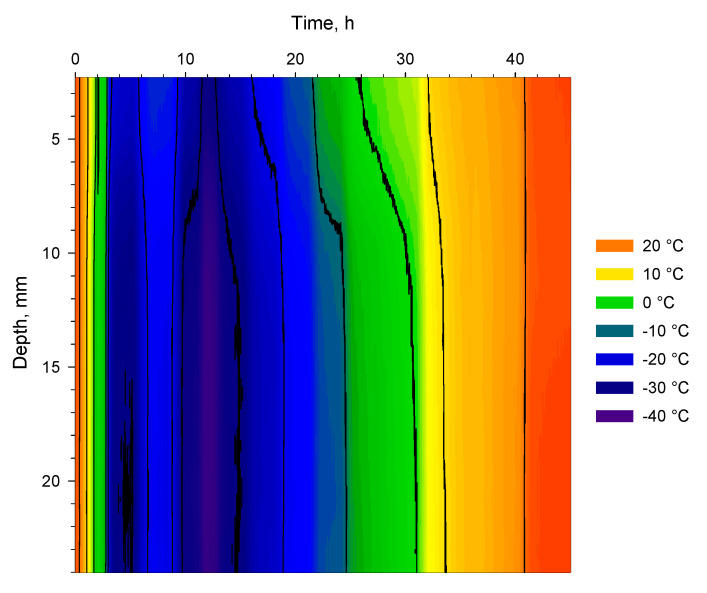
Full time/temperature profile from 0 to 45 h based on 2700 thermograms of Ge-cuvette along depth profile shown in Figure 4b. The main events of the freeze-drying cycle are visible: freezing, annealing, sublimation, and secondary drying. Black lines are 10 °C increments between −30 to +20 °C. Ambient pressure 0–11 h, 0.05 mbar 11–36 h, and 3 µbar 36–45 h. All temperature measurements were corrected with an emissivity of 0.9.

**Figure 7:**
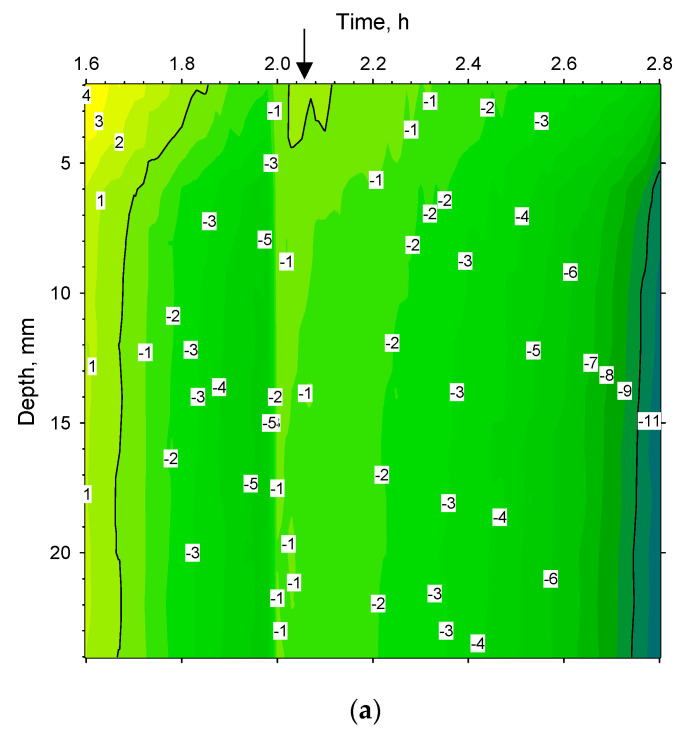

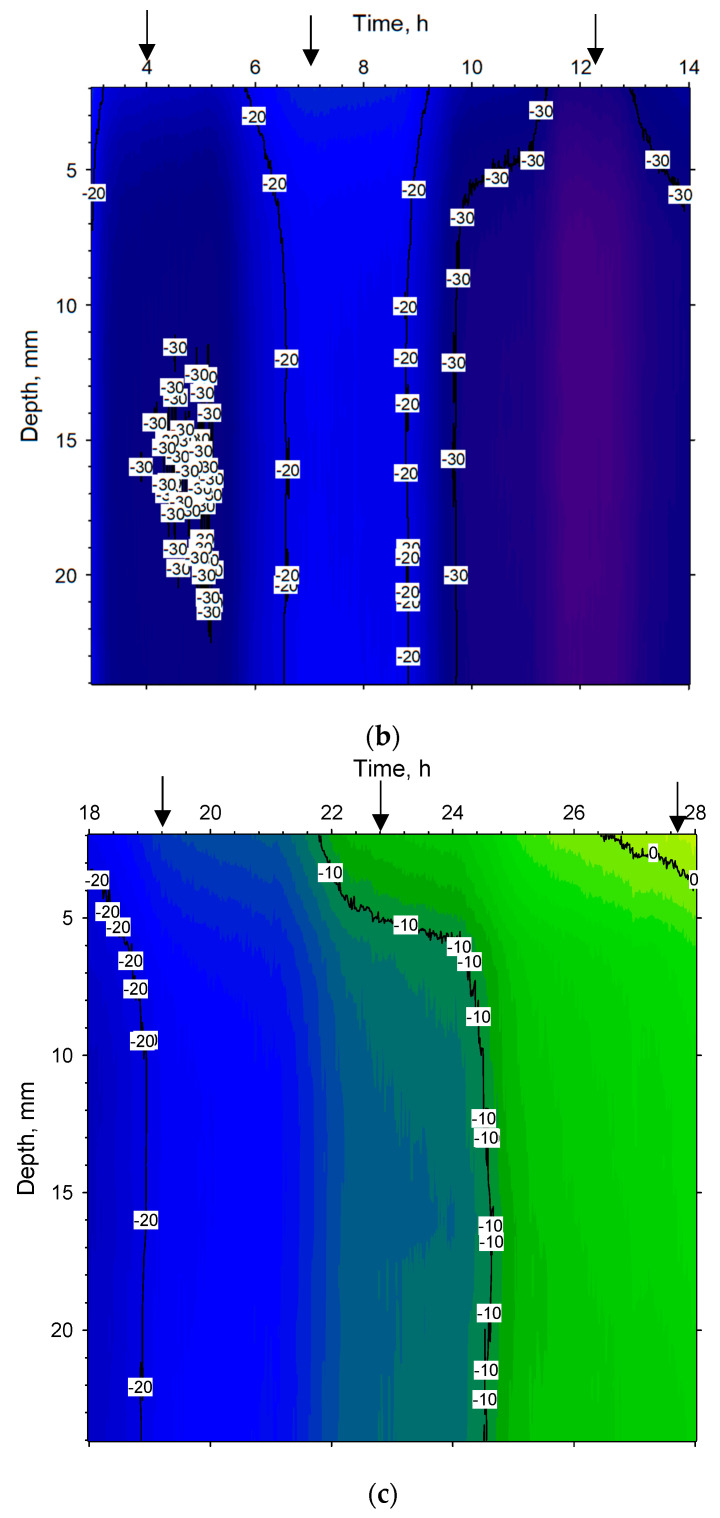

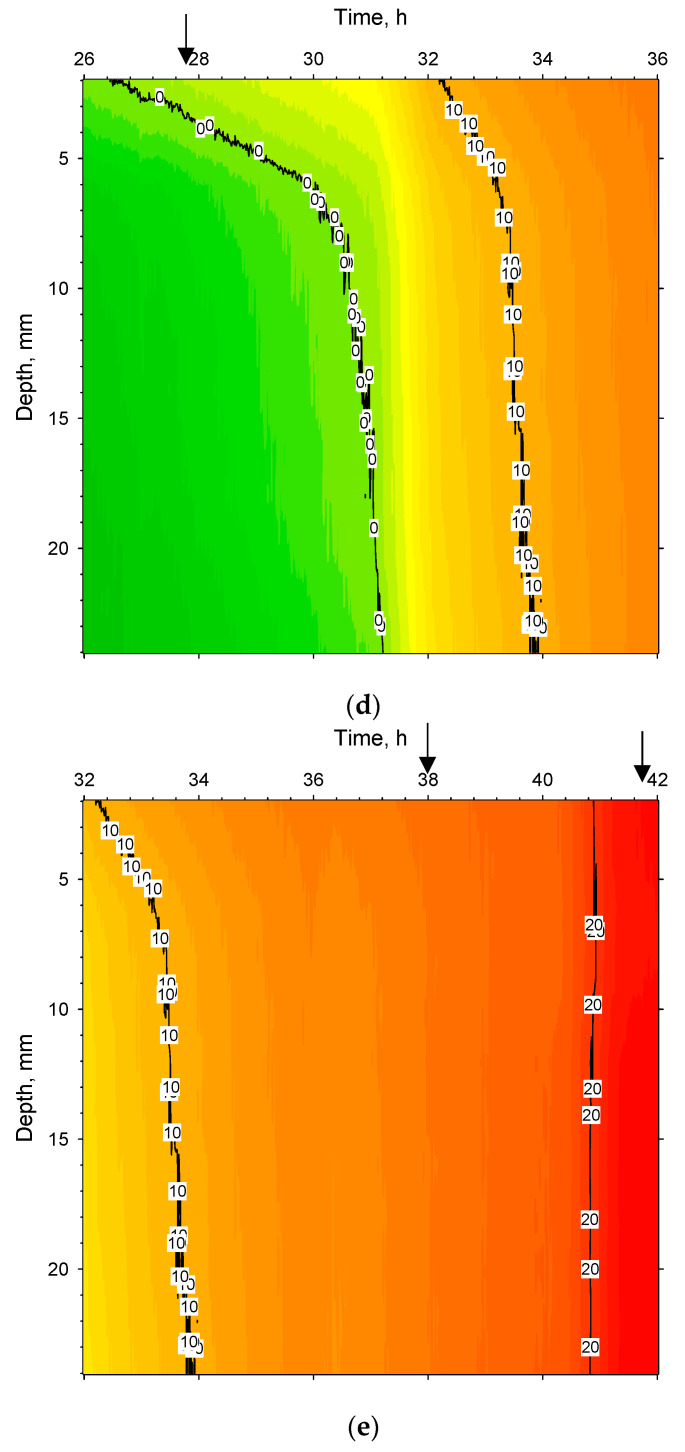
(**a**). Time/temperature profile from 1.6 to 2.8 h at ambient pressure based 72 thermograms of Ge-cuvette along depth profile shown in Figure 4b (freezing). Arrow at 2.05 h shows time-point of spatial contour plot for cuvette-side (shown in Figure 8a). Super-cooled liquid zone visible just before 2 h. (**b**). Time/temperature profile from 3 to 14 h based on 660 thermograms of Ge-cuvette along depth profile shown in Figure 4b, (freezing, annealing, and start of sublimation). Arrows at 4 h, 7 h, and 12.2 h show time points of spatial contour plot of the cuvette-side shown in Figure 8b–d, respectively. Ambient pressure from 3 h to 11 h and 0.05 mbar from 11–14 h. (**c**).Time/temperature profile from 18 to 28 h based on 600 thermograms of Ge-cuvette along depth profile shown in Figure 4b (sublimation). Arrows show positions at 19.2 h, 22.8 h and 27.8 h of spatial contour plot of the cuvette-side as shown Figure 8e–g, respectively. Pressure was 0.05 mbar from 18 to 28 h. (**d**).Time/temperature profile from 26 to 36 h based on 600 thermograms of Ge-cuvette along depth profile shown in Figure 4b (sublimation). Arrow shows position of spatial contour plot of the cuvette-side at 27.8 h (Figure 8g). Pressure was 0.05 mbar from 26 to 36 h. (**e**). Time/temperature profile based on 600 thermograms of Ge-cuvette along depth profile shown in Figure 4b from 32 to 42 h, (secondary drying). Arrows show positions at 38 h and 41.7 h of spatial contour plot of the cuvette side as shown in Figure 8h,i, respectively. Pressure was 0.05 mbar from 32 to 36 h and 3 µbar from 36 to 42 h.

**Figure 8:**
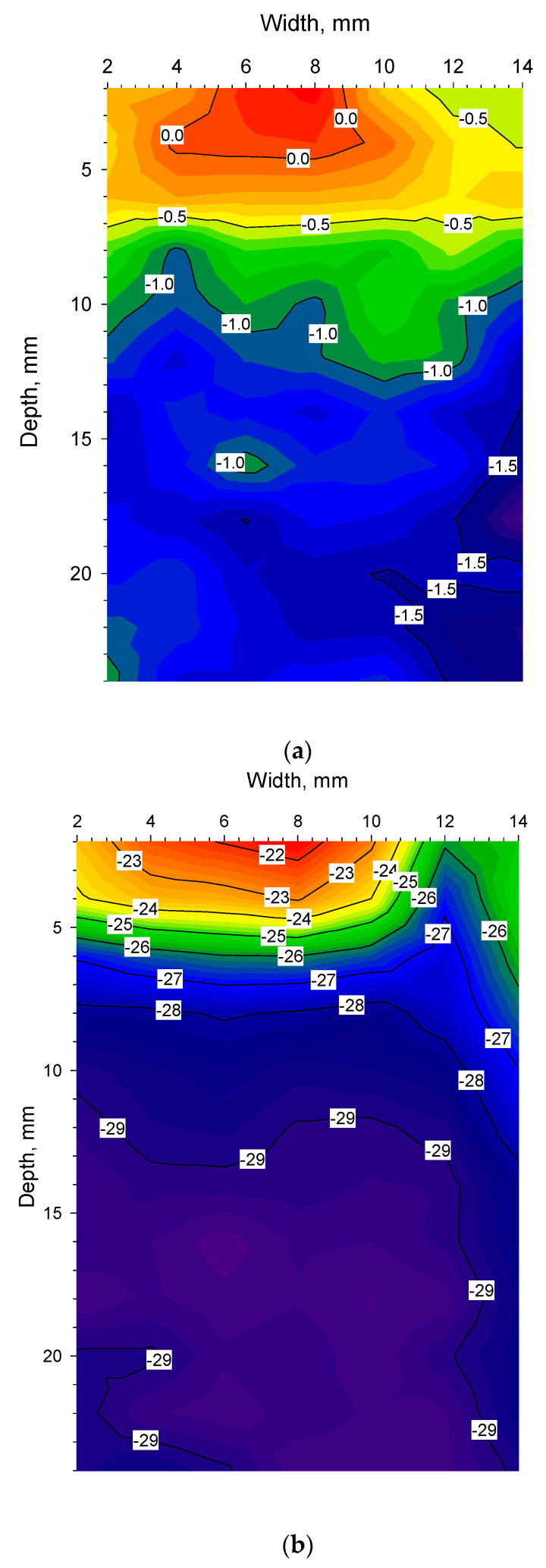

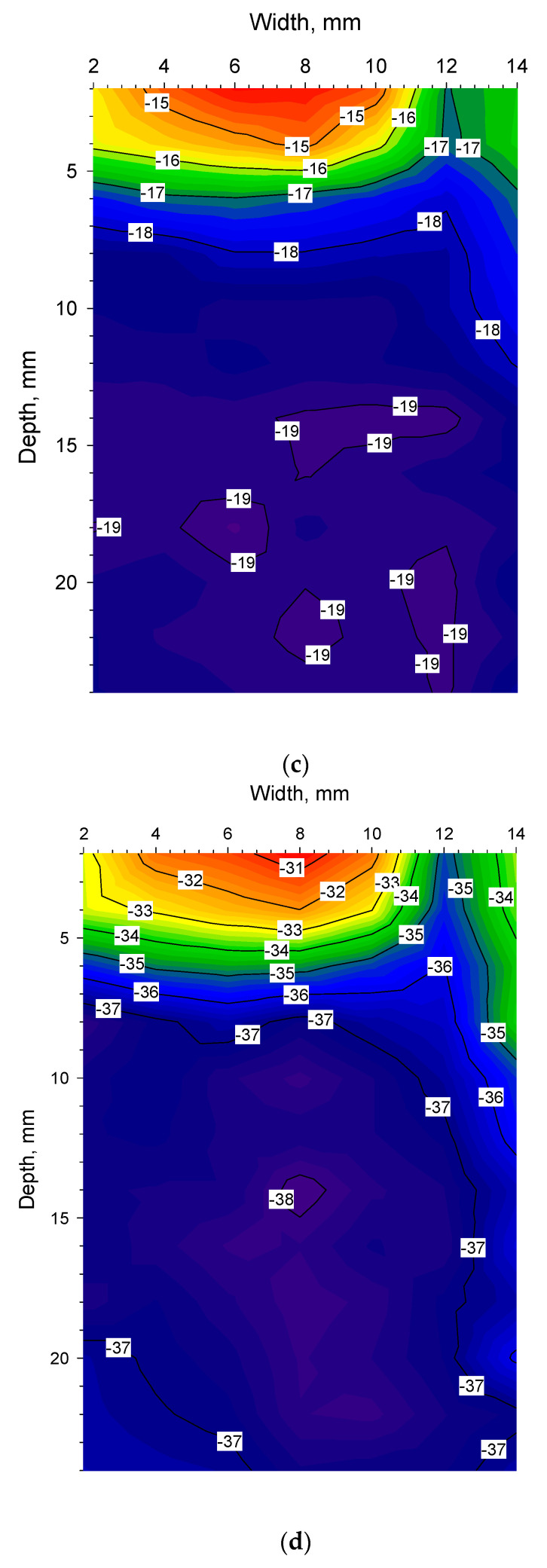

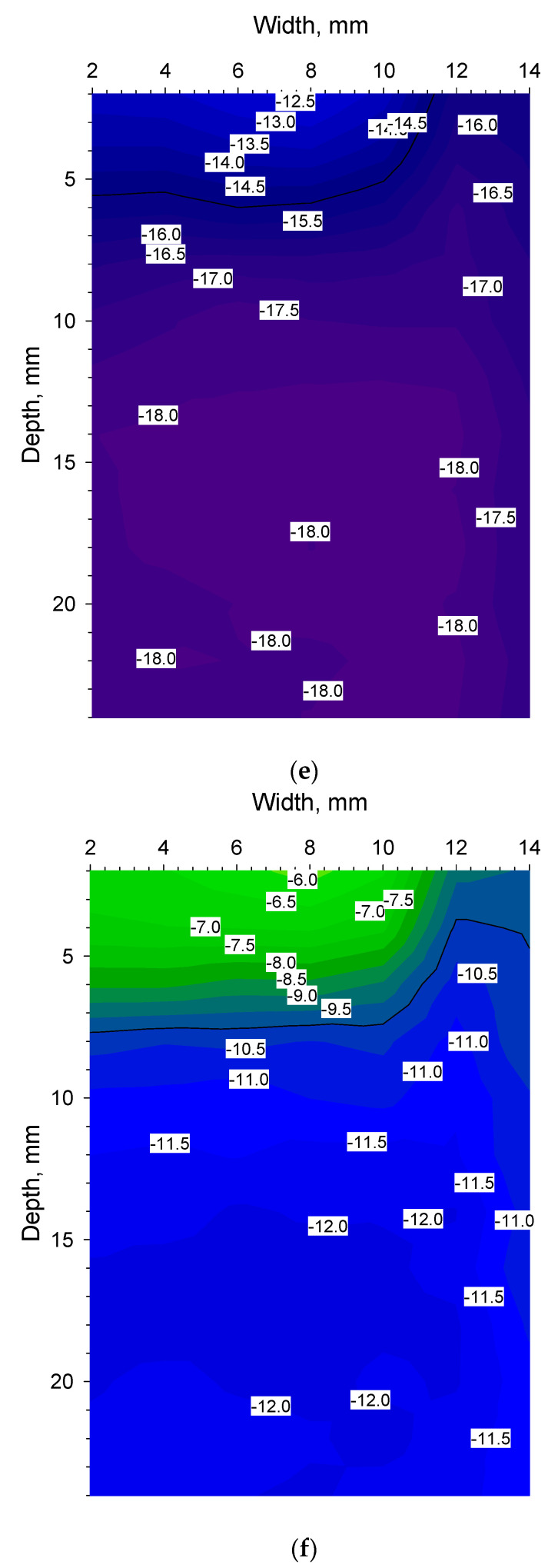

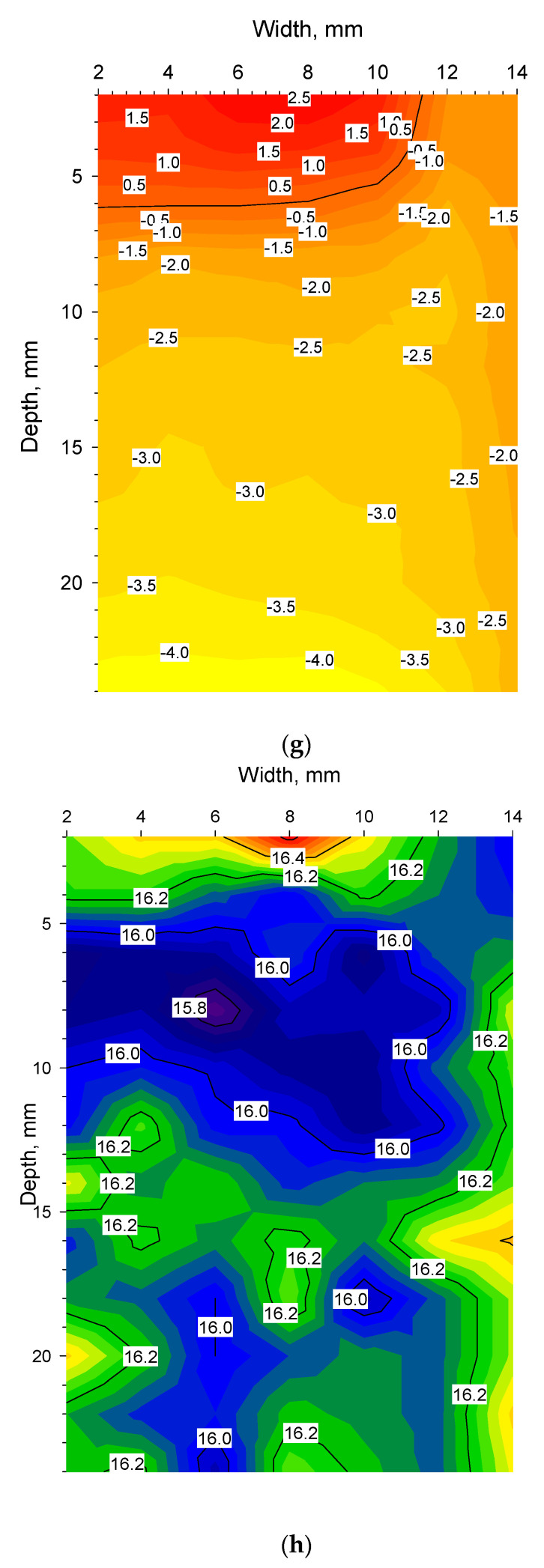

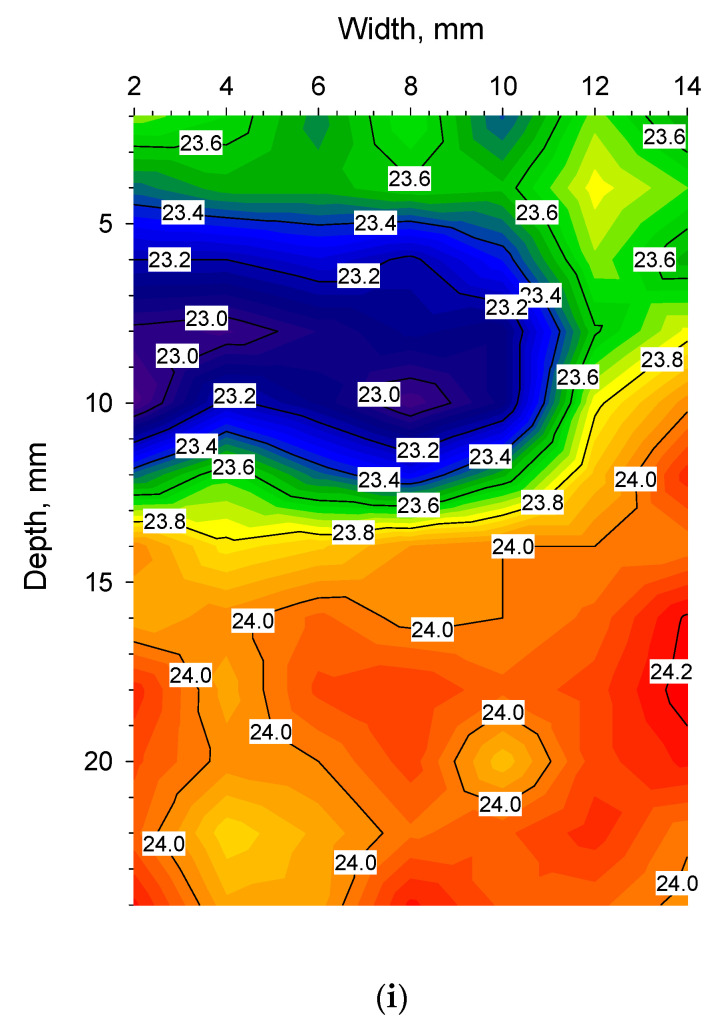
(**a**). Contour plot from 2.05 h displaying the freezing step where liquid is present at the surface. The temperature difference between top and bottom is maximum 1.5 °C. (**b**). Contour plot after 4 h, showing first freezing before annealing. Warmer zone still present at the top, up to 7 °C warmer than the bottom. (**c**). Contour plot after 7 h in the middle of the annealing step, where the top is 4 °C warmer than the rest of the material. The temperature is now 10 °C higher than after 4 h at the bottom of the cuvette. (**d**). Contour plot after 12.2 h during freezing where the material reached its lowest temperature of −38 °C. There is a 7 °C difference between central parts of the cuvette compared with the top. (**e**). Contour plot after 19.2 h in the sublimation step. Drying is taking place from the top downwards with a maximum difference of 5.5 °C between the top and bottom. Colour coding of temperature range −18.46 °C to 2.56 °C is the same for (**e**–**g**), e.g., when comparing the top of (**e**) with the bottom of (**f**), the light blue is about −12 °C in both figures. (**f**). Contour plot after 22.8 h in the sublimation step. The temperature has risen further and the difference is now 6 °C between the top and the bottom. Colour coding for temperature range −18.46 °C to 2.56 °C is the same for (**e**–**g**). (**g**). Contour plot after 27.8 h in the sublimation step. The temperature has risen above 0 °C at the top indicating that the ice has been removed. Further down in the material, ice is still present. The temperature difference is now 6.5 °C between the top and the bottom. Colour coding for temperature range −18.46 °C to 2.56 °C is the same for (**e**–**g**). (**h**). Contour plot after 38 h in secondary drying. Temperature difference is maximum 0.6 °C in the material. The material is 4 °C lower than the shelf temperature (set point 20 °C). (**i**). Contour plot after 41.7 h in secondary drying. The effect of the warm shelf can be seen (set point 30 °C). Colder area at the top could be indicative of evaporative cooling. In all other Figure 8a–h, the top is warmer than the bottom but at the end of the process, the situation is reversed. Temperature difference is 1 °C which is more than in (**h**).

In Figure 4a, the instrument setup is shown to give an overview of how the cuvettes were monitored inside the freeze dryer using the IR camera. The resulting reflection on the IR mirror and direct reading of the surface temperatures were recorded from above as already mentioned. In Figure 4b three cuvettes are shown with their respective measurement definitions, e.g., areas. Additionally, in one cuvette, a depth profile was evaluated through the complete freeze-drying cycle (labelled “profile” and placed centrally in the cuvette to the right in Figure 4b). The full freeze-drying cycle of 47 h is displayed in Figure 5a whereof the first 45 h were monitored using the infrared camera. The machine readings from the freeze dyer of shelf temperature, pressure, product temperatures, resistivity, and ice condenser temperature are shown in Figure 5a. The emissivity correction for all thermograms of 0.90 was deduced by matching the readout of the average shelf temperature for the large rectangle shown on top in Figure 4b (SHELF) in comparison with the actual shelf temperature readout from the freeze-dryer (separately checked with calibrated temperature sensors) shown in Figure 5a. That correction factor is identical to the one used in previous work [12]. The temperature recordings through the complete freeze-drying cycle of the 3 × 3 areas displayed in Figure 4b are shown in Figure 5b. That data can be directly compared with the temperature read-outs from the product temperature sensors (Pt100) shown in Figure 5a. Data obtained from the depth profile, the line shown in Figure 4b, are displayed in Figure 6 and Figure 7e for specific time windows corresponding to freezing, annealing, sublimation, and secondary drying. Spatial information was extracted from seven depth profiles placed next to each other as shown in Figure 4c resulting in 84 pixels (data points). Based on those 84 pixels, spatial contour plots could be made and are shown in Figure 8a–i. The specific time points for the spatial contour plots are indicated with vertical arrows in Figure 5a and in Figure 7a–e.

## 4. Monitoring of a Freeze-Drying Cycle for a Sucrose-Mannitol Solution

### 4.1. Full Freeze-Drying Cycle

Figure 6 shows the full freeze-drying cycle of the mannitol sucrose solution for the cuvette’s depth profile. Figure 6 is analogous to Figure 5a,b but contains more information. Supercooling takes place around 2 h before the material reaches 0 °C after which it ultimately solidifies and then undergoes an annealing step. After 11 h, at the end of the freezing step, the pressure is reduced and the material reaches a minimum temperature of −38 °C (at the bottom of the cuvettes) after approximately 12 h. Thereafter, the sublimation step starts with steady progress for 25 h. At the end of the sublimation phase, the pressure is reduced further and the secondary drying commences after 36 h lasting about 10 h. The colour coding for temperature in Figure 6 and Figure 7e, (i.e., different time windows) is directly comparable between the different figures as they are constructed from the same temperature/time data plot. For the spatial contour plots shown in Figure 8e–g (sublimation), it was possible to apply the same colour-coded temperature scale. However, for Figure 8a–d,h,i, the same colour-coded temperature scale could not be applied. Generally, too many details would be lost if the same temperature scale would be applied to all contour plots shown in Figure 8a–i.

### 4.2. Freezing and Super-Cooling

Figure 7a reveals the temperature distribution along the depth of the cuvette from 1.6 h to 2.8 h into the freeze-drying programme. Zero degrees is reached at the bottom of the cuvette 1.68 h into the programme and the surface reaches zero degrees ten minutes later at 1.84 h, as can be seen for the 0 °C isotherm. Thereafter, a sharp band of supercooled liquid (down to −5 °C) appears at 1.96 h. At 2.05 h, water reaches its freezing point at the surface until the material ultimately solidifies 0.1 h later. At this point, the lyoRX (resistivity sensor) increases rapidly to 95% indicating solidification as shown in Figure 5a. After about 2.8 h, −10 °C is reached up to about 5 mm below the surface. By inspecting the curvature of the isotherms, it can be seen that cooling is effected from the bottom upwards with an increasing cooling rate occurring after the actual freezing (narrower isotherm increments per unit of time). The arrow indicates the time point for the spatial contour plot shown in Figure 8a. Since freezing is such an important step in freeze-drying processes [11,19], controlled nucleation is a relatively new hardware and software feature that exists for some freeze-dryer models. In some configurations, an ice fog is blown over supercooled material [23,24]. Almost instant freezing can therefore be achieved in thousands of vials at the same time. This results in uniform ice crystals and improved product quality after sublimation. In these experiments, there was a time lag between different cuvettes at the time of freezing and controlled nucleation was not employed. The asymmetric temperature distribution during annealing (deep) freezing and sublimation was probably related to non-uniform freezing conditions.

### 4.3. Annealing

In Figure 7b subsequent freezing and annealing steps are shown from 3 to 14 h into the freeze-drying programme. The annealing step is included to obtain ice crystals of uniform and larger sizes that will improve the mass transfer of water vapour out of the material during sublimation [11]. The annealing step was not uniform in temperature since the top 5 mm was about 4 °C warmer than further down in the material. When observing the curvature (as a function of the position in the vial) for the −30 °C isotherm through the material, it can be seen that a slower cooling rate co-indices with the warmer zone from the annealing step. It is possible that this result is directly related to the size of the local ice crystals. The arrows indicate time points for the spatial contour plots shown in Figure 8b–d.

### 4.4. Sublimation

Figure 7c,d shows the sublimation step from 18 to 36 h into the freeze-drying programme. During sublimation, the chamber pressure was maintained at 0.05 mbar throughout. By observing the isotherms for −20, −10, and 0 °C in Figure 7c, it can be seen that the material dries from the top downwards. At 23 h, the colder core centrally in the material at −13 °C, can be seen from 12 to 17 mm depth. Interestingly, the −10 °C isotherm co-indices with the previous observations as described above for the annealing and deep-freezing steps. From 22 to 24 h into the programme, the drying progresses slowly. In Figure 7d, the same observation holds true for the 0 °C isotherm from 26 to 30 h into the programme. In the case of the 0 °C isotherm, the slower drying rate can be observed even deeper down in the material. As the material heats up above 0 °C the rate of drying increases in comparison with the 0 °C and −10 °C isotherms, especially when compared with the +10 °C isotherm. A more detailed study of the isotherms and curve-fitting based on a thermodynamic model is beyond scope of the current paper. The arrows indicate the time points for the spatial contour plots shown in Figure 8e–g as noted previously.

### 4.5. Secondary Drying

Figure 7e shows sublimation and secondary drying from 32 to 42 h into the freeze-drying programme. The secondary drying starts 36 h into the programme as the pressure was reduced to 3 µbar at that point. The arrows indicate the time points for the spatial contour plots shown in Figure 8h,i. It is interesting to note that in Figure 8h, the temperature in the material is next to uniform and around 16 °C from the bottom to the top. Whereas, in Figure 8i, the effect of the warmer shelf (set temperature of 30 °C) and the cooler upper part of the material could be indicative of evaporative cooling as the water leaves the drying material.

### 4.6. Residual Water Content in the Material

The residual water content in the material after drying was 4.15 ± 0.6% (*m*/*m*), *n* = 4, About 400 mg of solid sucrose mannitol was available per cuvette initially filled with 8 mL of the 5% pharmaceutical formulation. Two replicates per cuvette could be measured with sufficient accuracy using Karl Fischer titration. It is possible that some mannitol hydrate was present in the final material leading to the somewhat high water content [11]. However, this study was not performed to optimize the drying process to achieve an extremely dry final material.

## 5. Conclusions

Infrared thermography provides superior spatial and thermal resolution when monitoring a freeze-drying cycle in contrast to traditional means such as Pt-100 probes. In this work, this feature was further exploited by using cuvettes with one side made of germanium. The correct choice of material in the vial/cuvette wall is important to achieve sufficient thermal and spatial resolution to record very small temperature differences inside the material that is drying. In this work, the full depth profiles of temperature could also be recorded thanks to this development. The isotherms show different rates of temperature change per mm of material as a function of time during freezing, sublimation, and secondary drying. These slopes also differ depending on the depth in the material. These effects are derived from different dynamics in different parts of the freeze-drying process. The dynamics of freezing and annealing have an impact on the subsequent sublimation phase. There was also some degree of asymmetry in the temperature distribution in the annealing step that could be observed throughout the sublimation step. Thanks to the high thermal resolution of this set-up and the geometry of the measurement capability, it was possible to detect relatively cooler upper layers in the material seen at the end of the secondary drying. This effect could be related to evaporative cooling. 

Newer IR cameras come with an increased number of pixels of their micro-bolometers, which should make studies that are even more detailed possible with higher resolution, especially in combination with appropriate lenses. Data transfer and temperature control of the germanium lens in the IR camera and its microbolometer is easier if the IR camera is kept outside the freeze-drying chamber. No detailed study was made to compare cuvettes equipped with a Pt-100 probe with cuvettes that had no Pt-100 probe. No striking differences between the thermograms recorded for these two categories of cuvettes were observed. This suggests that the effect on the temperature distribution in the presence of probes is relatively small but it could now be investigated further thanks to the high thermal and spatial resolution shown here. This new development consequently provides a much sharper tool for these kinds of studies. 

## Figures and Tables

**Table 1:** Physical properties of germanium (HEAR/HEAR coated) and Schott B 270 superwite modified soda-lime glass used in the cuvettes.

Physical Property	Germanium Umicore ^1^	Optical Glass B270 Superwite ^2^	Unit
Thermal conductivity	60.2	0.92	W/(m K)
Specific heat	0.31	0.86	J g^−1^ K^−1^
LWIR transmission, T = I/I_0_ for 7–14 µm	97	Negligible ^3^	%
LWIR reflectance	0.69	no info	%

^1^ Data obtained from Umicore Electro-Optic Materials. ^2^ Data obtained from Schott DESAG https://www.schott.com/en-bg/products/b-270-p1000313/technical-details (accessed on 3 February 2022). ^3^ Shown experimentally in Figure 1b.

## Data Availability

Not applicable.
